# Circulating Exosomal miR-150-5p and miR-99b-5p as Diagnostic Biomarkers for Colorectal Cancer

**DOI:** 10.3389/fonc.2019.01129

**Published:** 2019-10-23

**Authors:** Ya jing Zhao, Xingguo Song, Limin Niu, Youyong Tang, Xianrang Song, Li Xie

**Affiliations:** ^1^School of Medicine and Life Sciences, University of Jinan, Shandong Academy of Medical Sciences, Jinan, China; ^2^Department of Clinical Laboratory, Shandong Cancer Hospital and Institute, Shandong First Medical University and Shandong Academy of Medical Sciences, Jinan, China; ^3^Shandong Provincial Key Laboratory of Radiation Oncology, Shandong Cancer Hospital and Institute, Shandong First Medical University and Shandong Academy of Medical Sciences, Jinan, China

**Keywords:** exosomes, colorectal cancer, miR-99b-5p, miR-150-5p, diagnosis

## Abstract

**Background:** Circulating exosomal miRNAs are potential non-invasive biomarkers for colorectal cancer. The present study aimed to validate the novel sensitive and specific exosomal miRNA biomarkers for diagnosing colorectal cancer (CRC).

**Patients and Methods:** Exosomes isolated from the serum of CRC patients and healthy donors by ultracentrifugation were characterized using TEM, qNano, and immunoblotting. The exosomes from 2 healthy donors and 4 CRC patients were subjected to RNA isolation and miRNA sequencing. The differently expressed miRNAs from 165 primary CRC patients and 153 healthy donors were substantiated by RT-qPCR.

**Results:** The RNA-sequence data analysis revealed that 29 exosomal miRNAs (20 downregulated and 9 upregulated) with >1.5-fold difference between CRC patients and healthy donors were selected. The serum exosomal miR-99b-5p and miR-150-5p levels were significantly downregulated in CRC patients as compared to healthy donors (*p* < 0.0001 and *p* < 0.0001, respectively) and benign disease (*p* = 0.009 and *p* < 0.0001, respectively). The expression levels of exosomal miR-99b-5p and miR-150-5p were significantly decreased in early CRC patients as compared to healthy donors (*p* < 0.0001 and *p* < 0.0001, respectively). The expression levels of exosomal miR-99b-5p and miR-150-5p were significantly increased postoperatively (*p* = 0.0058 and *p* < 0.0001, respectively).

**Conclusions:** The present study demonstrated that serum exosomal miRNAs are promising, sensitive, specific, and non-invasive diagnostic biomarkers for CRC.

**Impact:** This is the first study to specifically identify exosomal miR-99b-5p and miR-150-5p associated with CRC. This study, therefore, might deepen the understanding of tumor-derived exosomes for CRC diagnosis.

## Introduction

Colorectal cancer (CRC) is the third most common cancer worldwide with 1.36 million new cases annually and about 700 thousand deaths (1, 2). Approximately, 50% of CRC patients are deceased as a consequence of late detection of advanced disease with localized or distant metastases (3). These phenomena highlight and underscore a need for the identification and development of robust and inexpensive screening biomarkers that are non-invasive and facilitate the early detection of CRC such as fluid biopsy based on blood contents including cell-free DNA (4), circulating tumor cells (5), and exosomes (6).

Exosomes are known as extracellular vesicles, with a diameter of 50–150 nm, released from different cell types (7–9), which are regarded as critical mediators of intercellular communications including the delivery of biological signals and selective cargo between different cells, thereby regulating multiple biological procedures (10, 11). Exosomes also exert pleiotropic roles in cancer progression, metastasis, immune modulation, and drug resistance (12–16). The critical discovery of exosome-mediated communication is the transfer of genetic information via exosomes, such as messenger RNAs (mRNAs) and short non-coding microRNAs (miRNAs), to neighboring cells, or distant organs. For instance, miRNAs in cancer exosomes are hormones, which are vital in mediating cancer progression and metastasis, thus emerging as promising biomarkers for cancer (17).

miRNAs are short (20–24 nt), single-stranded, non-coding, and evolutionarily conserved RNA molecules, which regulate the gene expression in a post-transcriptional manner (18). These miRNAs have been found to be associated with cancer progression, proliferation, and migration (19, 20). The exosomal miRNAs are existed stably in the blood of cancer patients. Emerging evidence suggested that these miRNAs can mediate cell-to-cell communication, thereby affecting carcinogenesis, metastasis, and relapse; thus, exosomal miRNAs could be used as molecular biomarkers for cancer. Recent studies have shown that serum exosomal miR-1290 was significantly overexpressed as compared to the healthy controls and can discriminate patients from those with malignancies of other histological types in high-grade serous ovarian carcinoma (21). The level of serum exosomal miR-210 was upregulated at an advanced stage with potential as a novel non-invasive biomarker for the detection and prognosis of clear cell renal cell carcinoma (22). The exosomal miR-223-3p levels of the patients with breast cancer were significantly increased as compared to the healthy controls (23). Exosomal miR-106a influenced the survival and promoted the tumorigenesis by regulating several pathways, which are valuable for the diagnosis and prognosis of hepatocellular carcinoma (24).

In this study, we utilized small RNA sequencing and quantitative PCR to search for the differential exosomal miRNAs between CRC patients and healthy donors, respectively. Consequently, exosomal miR-150-5p and miR-99b-5p were selected and their correlation with clinical characteristics and diagnostic efficiency for CRC diagnosis was analyzed; thus, these served as novel biomarkers for CRC.

## Materials and Methods

### Patients

A total of 169 CRC patients, 155 healthy donors, and 20 benign disease patients were admitted in the Shandong Cancer Hospital from September 2017 to July 2018. Exosomes from 2 healthy donors and 4 CRC patients were subjected to miRNA sequencing, and 165 CRC patients, exosomes from other 155 healthy donors, and 20 benign disease patients were subjected to qPCR verification. Written informed consent was obtained from all subjects. Tumor staging was estimated according to the AJCC Cancer Staging Handbook of the American Joint Committee on Cancer, 2010. All patients did not receive the anti-tumor treatment before serum collection or suffered from any other endocrine, immune, or metabolic diseases. Sera were collected from 20/165 patients with CRC, who underwent surgery after 2 months. Patient characteristics and history of diabetes of CRC patients are shown in **Table 3**.

### Isolation of Exosomes

The exosomes were isolated using ultracentrifugation as described previously (25). Briefly, serum underwent centrifugation at 10,000 × *g* for 30 min at 4°C to remove the cellular debris, followed by ultracentrifugation (Beckman Coulter, Brea, CA, USA) at 100,000 × *g* for 2 h at 4°C for exosome precipitation. Then, the exosome sediment was analyzed by transmission electron microscopy (TEM), qNano, and immunoblotting, miRNA sequencing, and real-time PCR.

### TEM Assay

TEM was performed to identify the purified exosomes. The exosome pellets were transferred to the grids in a 50 μL drop of 1% glutaraldehyde for 5 min and transferred to a 100-μL drop of distilled water and let the grids stand for 2 min. Then, the grids were placed directly to a 50-μL drop of uranyl-oxalate solution (pH 7), for 5 min and covered with a parafilm-covered glass dish covered anon ice. Subsequently, the grids were washed seven times with distilled water for 2 min each and examined using a JEM-1200EX transmission electron microscope (JEOL, Japan) operated at 100 kV.

### Tunable Resistive Pulse Sensing (TRPS)

The size of the nanoparticle was measured using TRPS and on the qNano (Izon Science Ltd, Christchurch, New Zealand). Data were analyzed using Izon Control Suite v.3.3.2.2000 (Izon Science Ltd.).

### Immunoblotting

An equivalent amount of exosomal or cellular proteins was resolved by SDS-PAGE and transferred to PVDF membranes (Millipore, Billerica, MA, USA). The membranes were blocked with 5% milk in Tris-buffered saline containing 0.1% Tween 20 (TBST) for 1 h and probed overnight at 4°C with rabbit primary antibodies against CD63, TSG101, and GM130, followed by incubation with to HRP-conjugated secondary antibodies (Proteintech) for 1 h at room temperature. The immunoreactive bands were visualized using ECL blotting detection reagents (Bio-Rad, USA), and developed and fixed onto X-ray films.

### Differential miRNA Expression of RNA-Sequence Data Sets and Analysis

A total of 3 μg RNA from each sample was used as input material for the generation of small RNA library. Following cluster generation, the libraries were sequenced on an Illumina HiSeq 2500/2000 platform (Illumina, USA), and 50-bp single-end reads were generated. After sequencing, the data were subjected to the following preliminary analyzes and procedures, which were performed by the Novogene Corporation: quality control analysis, read mapping to the homo spaiens genome, transcriptome assembly, coding potential analysis, conservative analysis, target gene prediction, gene expression level quantification, differential expression analysis, and Kyoto Encyclopedia of Genes and Genomes (KEGG) enrichment analysis. Differential expression analysis of two conditions/groups was performed using the DESeq R package (1.8.3). The *P*-values was adjusted using the Benjamini& Hochberg method. Corrected *P*-value of 0.05 was set as the threshold for significantly differential expression by default. We used KOBAS software to test the statistical enrichment of the target gene candidates in KEGG pathways. GOseq based Wallenius non-central hyper-geometric distribution, which could adjust for gene length bias, was implemented for GO enrichment analysis. Predicting the target gene of miRNA was performed RNAhybrid: (https://bibiserv.cebitec.uni-bielefeld.de/rnahybrid/); PITA: (genie.weizmann.ac.il/pubs/mir07/mir07_dyn_data.html); miRanda: (http://www.microrna.org/microrna/getMirnaForm.do) (Table S1).

### RNA Isolation and Real-Time PCR

Total RNAs were extracted by TRIzol reagent (Thermo Fisher Scientific, Carlsbad, CA, USA) according to the manufacturer's instructions. The extracted RNA was reverse-transcribed into complementary DNA (cDNA) using the Mix-X miRNA First-Strand Synthesis Kit (TaKaRa Bio, Nojihigashi, Kusatsu, Japan) according to the manufacturer's instructions. Real-time PCR was performed using TB-Green Premix Ex Taq II Reagent (TaKaRa Bio) according to the manufacturer's instructions. U6 was used as an internal control (26). Each sample was analyzed in duplicate. The PCR reaction was evaluated by melting curve analysis. The relative quantification of miRNA expression was evaluated using the ΔCT method (Ct^miRNA^-Ct^U6^) as described previously (27).

### Statistical Analysis

Statistical analysis was performed using SPSS 22.0 (IBM, Ehningen, Germany) and GraphPad Prism 6.0 (San Diego, CA, USA). The comparisons were performed using Mann–Whitney *U* or *t*-test, and the difference between paired values was evaluated using a paired *t*-test. Multiple comparisons were performed using one-way analysis of variance (ANOVA) or Kruskal–Wallis one-way ANOVA. Receiver operating characteristic (ROC) curves with the corresponding C statistics (area under the curve, AUC), based on the logistic models, were used to determine the corresponding cutoff points with the pathological diagnosis treated as the “gold standard.” *p* < 0.05 was considered to be statistically significant. In addition, each miRNA or the combination of miRNAs were distinguished by analysis.

## Results

### Identification of Isolated Exosomes

**Figure 1:**
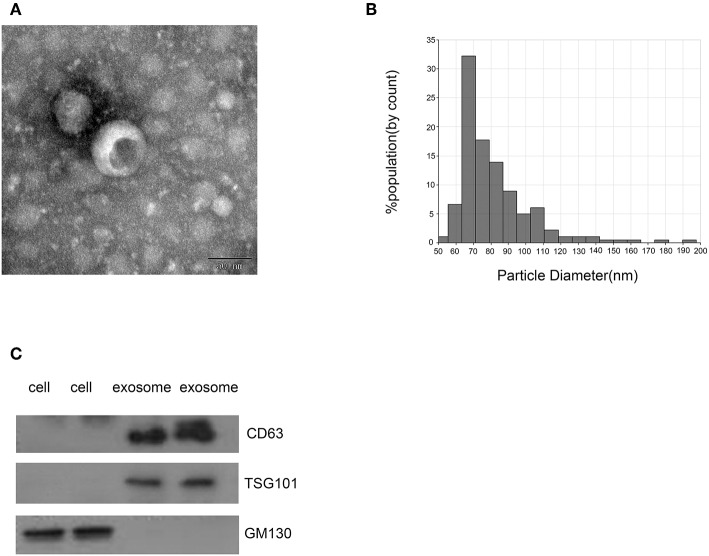
Identification of isolated exosomes. **(A)** TEM image showed representative data of exosomes from CRC patients with 50–150 nm diameter of the tumors (scale bar: 50 nm; high voltage (HV) = 100 kV). **(B)** Distribution of exosomes with 50–150 nm diameter; the samples were obtained from CRC patients based on the qNano system. **(C)** Western blot analysis of CD63, TSG101, and GM130 as exosomal markers.

Exosomes isolated from the sera of CRC patients and healthy donors by ultracentrifugation were characterized using TEM, qNano, and immunoblotting (Figure 1A) illustrates the typical exosome-like round morphology with 50–150 nm diameter by TEM, which was in agreement with the result from qNano (Figure 1B). In addition, CD63 and TSG101, two well-known protein markers (28, 29), are enriched in exosomes from CRC patients but are undetectable in the cell (Figure 1C). On the other hand, GM130 is a tethering factor associated with giantin in the cis-Golgi compartment commonly used as a negative control for exosome (Figure 1C) (30). It was only detected in the cell but not in CRC exosome. Therefore, these results confirmed that the vesicles isolated by ultracentrifugation were exosomes.

### Exosomal miRNA Profile of the CRC Patients

**Figure 2:**
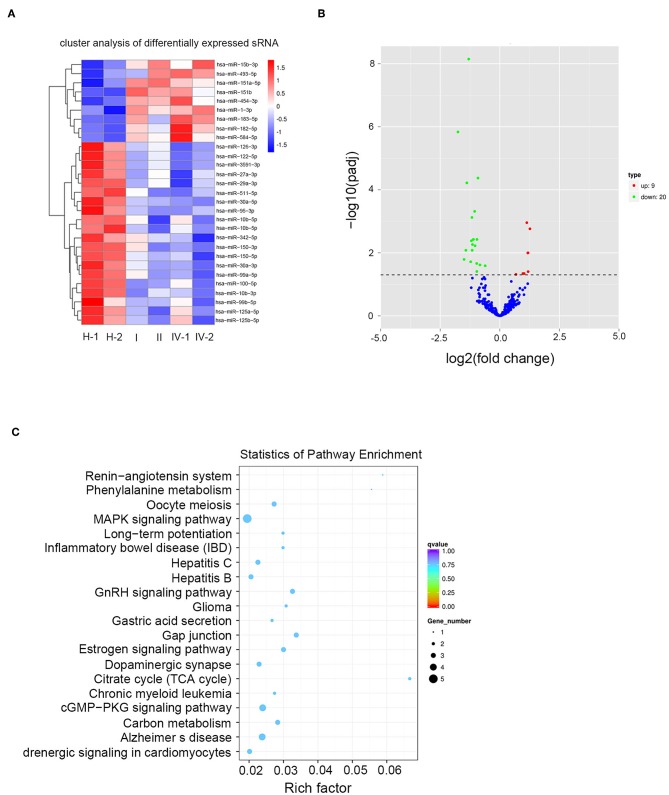
Exosomal miRNA profile of the CRC patients. **(A)** A heat map was generated after supervised hierarchical cluster analysis. The differential miRNA expression is shown in red (upregulation) vs. blue (downregulation) (*p* < 0.05). **(B)** Volcano plot compared the expression fold-change of exosomal miRNA in CRC patients vs. healthy donors. The red dots represent the upregulated miRNA, and the green dots represent the downregulated miRNA. **(C)** Candidate target gene KEGG enrichment analysis.

Exosomes from 2 healthy donors and 4 CRC patients were subjected to RNA isolation and miRNA sequencing. A total of 1,145 differential miRNAs were screened between the healthy donors and CRC patients via analyzing the raw miRNA expression profiling data (Figure 2A). Moreover, 29 miRNAs (20 downregulated and 9 upregulated, Tables 1, 2) were selected based on >1.5-fold difference between the two groups (Figure 2B). KEGG analysis demonstrated that the target genes of differently expressed miRNAs were primarily assigned to 20 pathways, among which, mitogen-activated protein kinase (MAPK) and cyclic guanosine monophosphate-protein kinase G (cGMP-PKG) signaling pathways were mainly involved in exosomal miRNA functions in CRC (Figure 2C).

**Table 1:** Down-regulated miRNA of CRC patients.

**miRNA**	**Fold change**	***P*-value**	**Description**
hsa-miR-122-5p	−3.3755	1.28E-08	Down
hsa-miR-3591-3p	−3.3753	1.28E-08	Down
hsa-miR-511-5p	−2.8280	0.0009006	Down
hsa-miR-150-3p	−2.6818	0.0003886	Down
hsa-miR-150-5p	−2.6076	8.83E-07	Down
hsa-miR-30a-5p	−2.4679	2.09E-11	Down
hsa-miR-342-5p	−2.3353	0.001128	Down
hsa-miR-10b-3p	−2.2874	0.0001459	Down
hsa-miR-99a-5p	−2.2484	1.55E-05	Down
hsa-miR-99b-5p	−2.2347	0.0003671	Down
hsa-miR-95-3p	−2.2156	0.0002078	Down
hsa-miR-125a-5p	−2.1529	0.0001209	Down
hsa-miR-30a-3p	−2.0763	8.51E-06	Down
hsa-miR-29a-3p	−2.0544	0.0002452	Down
hsa-miR-125b-5p	−1.9581	0.001341	Down
hsa-miR-10b-5p	−1.9510	0.0027488	Down
hsa-miR-126-3p	−1.9419	0.000115	Down
hsa-miR-100-5p	−1.8859	4.98E-07	Down
hsa-miR-27a-3p	−1.7803	0.0015575	Down
hsa-miR-146b-5p	−1.5288	0.0017378	Down

**Table 2:** Up-regulated miRNA of CRC patients.

**miRNA**	**Fold change**	***P*-value**	**Description**
hsa-miR-151a-5p	2.4013	4.57E-05	Up
hsa-miR-454-3p	2.2795	0.0028966	Up
hsa-miR-183-5p	2.2725	0.0004991	Up
hsa-miR-182-5p	2.590	0.0005314	Up
hsa-miR-1-3p	2.1931	2.59E-05	Up
hsa-miR-493-5p	2.2099	0.0037373	Up
hsa-miR-15b-3p	1.9826	0.0035878	Up
hsa-miR-151b	1.9599	0.0035665	Up
hsa-miR-584-5p	1.5983	0.0041064	Up

### Characterization of Identified Two Serum Exosomal miRNAs

**Figure 3:**
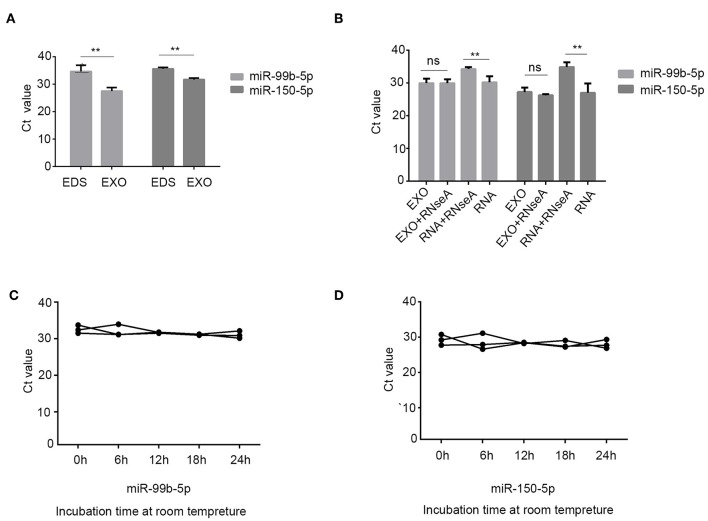
Characterization of identified two serum exosomal miRNAs. **(A)** Expression levels of miR-99b-5p and miR-150-5p from serum exosome (EXO) and exosome-depleted supernatant (EDS). **(B)** qRT-PCR analysis of the two miRNAs in the exosomes or isolated nucleic acids treated with RNase A. **(C,D)** The expressions of the two serum exosomal miRNAs when incubated at room temperature (**p < 0.01, ns, not siginificant).

To confirm whether serum miRNAs were exclusively distributed into exosomes. We compared the expression of two miRNAs between exosome-depleted supernatant and exosomes. The results demonstrated that the expression of miR-99b-5p and miR-150-5p in exosomes were increased compare with those in EDS, which were distributed mainly in exosomes (Figure 3A). In addition, we validated the stability of exosomal miRNAs. The expression of miRNAs in exosomes were still unchanged upon RNase A treatment (Figure 3B). In brief, the results show that miR-99b-5p and miR-150-5p mainly exist in exosomes, which protect miRNAs from enzyme treatment. In room temperature incubation test, the exosomes were maintained at room temperature for 0, 6, 12, 18, 2 4 h. No significant changes were found for the expression levels of miR-99b-5p and miR-150-5p at different time-points (Figures 3C,D).

### Exosomal miR-99b-5p and miR-150-5p as Biomarkers for CRC

**Figure 4:**
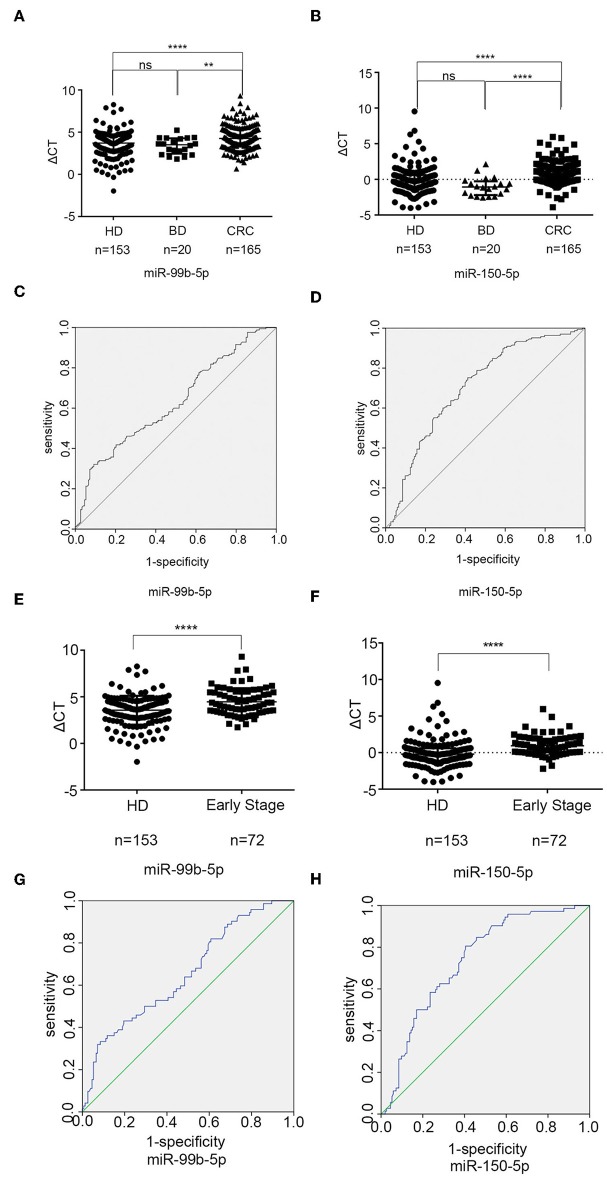
Exosomal miR-99b-5p and miR-150-5p as biomarkers for CRC. **(A,B)** The scatter plot compared the exosomal miR-99b-5p and miR-150-5p levels in the serum of healthy donors (*n* = 153), patients with benign diseases (*n* = 20), and CRC patients (*n* = 165) (**p < 0.01, ****p < 0.0001). **(C)** The AUC of serum exosomal miR-99b-5p was 0.628 in 165 CRC patients and 153 healthy donors. **(D)** The AUC of serum exosomal miR-150-5p was 0.707 in 165 CRC patients and 153 healthy donors. **(E,F)** The scatter blot compared the exosomal miR-99b-5p and miR-150-5p levels in healthy donors (*n* = 153) with early CRC patients (*n* = 72) (****p < 0.0001). **(G)** The AUC of serum exosomal miR-99b-5p was 0.652 in 72 early CRC patients and 153 healthy donors. **(H)** The AUC of serum exosomal miR-150-5p was 0.736 in 72 early CRC patients and 153 healthy donors.

To identify the potential exosomal miRNA biomarkers in CRC, differently expressed two miRNAs were subjected to qPCR for large-scale validation using samples from 165 CRC patients and 153 healthy donors. As shown in Figures 4A,B, the levels of exosomal miR-99b-5p and miR-150-5p were significantly decreased in CRC patients (*p* < 0.0001 and *p* < 0.0001, Kruskal–Wallis one-way ANOVA, respectively) as compared to healthy donors as well as benign patients (*p* = 0.009 and *p* < 0.0001, Kruskal–Wallis one-way ANOVA, respectively), whereas no significant differences in these two miRNAs were observed between healthy donors and benign patients (*p* = 0.814 and *p* = 0.063, Kruskal–Wallis one-way ANOVA, respectively).

In addition, we also analyzed the correlation between miR-99b-5p, miR-150-5p, and clinicopathological characteristics (Table 3 and Table S1). These factors were not related to patients' age, histological type, lymph node metastasis status, and pathological stage but related to the gender (*p* = 0.019, *t*-test and *p* = 0.002, Mann–Whitney *U-*test, respectively).

**Table 3:** Characteristics of CRC patients for differentially expressed exosomal miR-99b-5p and miR-150-5p.

**Characteristic**	**No. cases**	**miR-99b-5p**		**miR-150-5p**	
		**Median with interquartile range**	***P*-value**	**Median with interquartile range**	***P*-value**
**Age (year)**					
<61	80	4.4925 (4.132–4.7335)	0.32	0.7100 (0.5593–1.2125)	0.944
≥61	85	4.035 (3.9589–4.6436)		0.8395 (0.5322–1.2309)	
**Gender**					
Male	110	4.5175 (4.2584–4.8499)	0.019	1.055 (0.8336–1.4138)	0.002
Female	55	3.865 (3.6622–4.3111)		0.2450 (0.0832–0.8241)	
**Drinking status**					
Yes	31	4.0550 (3.7801–4.9247)	0.998	0.5800 (0.1881–0.9613)	0.183
No	134	4.300 (4.1042–4.6020)		0.7700 (0.7079–1.2540)	
**Diabetes status**					
Yes	21	4.675 (3.6963–4.9389)	0.92	0.495 (−0.0834–1.4948)	0.571
No	144	4.21 (4.1220–4.6148)		0.7375 (0.6875–1.1773)	
**Tumor position**					
Rectum	102	4.1025 (3.9865–4.5526)	0.276	0.63 (0.5015–1.0566)	0.087
Colon	63	4.3800 (4.1446–4.9102)		1.06 (0.6806–1.5127)	
**Tumor size**					
<30	52	4.4925 (3.8953–4.7947)	0.926	0.3525(0.2954–1.1800)	0.241
≥30	63	4.1900 (4.0147–4.7632)		1.050(0.7042–1.4445)	
Unknown	50				
**Lymph node metastasis status**					
Yes	88	4.2675 (3.986–4.6219)	0.629	0.6400 (0.4981–1.1705)	0.356
No	74	4.210 (4.0896–4.7637)		0.9275 (0.6361–1.295)	
Unknown	3				
**Distant metastasis**					
Yes	37	4.5200 (3.8842–4.978)	0.862	0.715 (0.5686–1.6924)	0.844
No	128	4.210 (4.1002–4.5993)		0.7550 (0.5788–1.0888)	

To evaluate the diagnostic performance of miR-99b-5p and miR-150-5p for CRC, a ROC curve was calculated. As shown in Figures 4C,D, the AUC of miR-99b-5p was 0.628 [95% confidence interval (CI): 0.567–0.689] with 32.1% sensitivity and 90.8% specificity, the cut off was 5.0225, while the AUC of miR-150-5p was 0.707 (95% CI: 0.649–0.764) with 75.2% sensitivity and 58.8% specificity, the cut off was −0.0325. Taken together, the current data supported that exosomal miR-99b-5p and miR-150-5p acted as biomarkers for CRC.

Furthermore, to elucidate the early CRC diagnosis efficacy of exosomal miR-99b-5p and miR-150-5p, we analyzed early CRC patients (31) (I = 16, II = 56), and healthy donors (*n* = 153). As shown in Figures 4E,F, expression levels of exosomal miR-99b-5p and miR-150-5p were significantly decreased in early CRC patients as compared to the healthy donors (*p* < 0.0001 and *p* < 0.0001, Mann–Whitney *U-*test, respectively). Next, ROC curves were employed to evaluate the performance of exosomal miR-99b-5p and miR-150-5p as serum biomarkers for the diagnosis of early CRC. Figures 4G,H show that the AUC of miR-99b-5p was 0.652 (95% CI: 0.576–0.729) with the sensitivity of 33.3% and the specificity of 91.5%, the cut off was 5.0975, while the AUC of miR-150-5p was 0.736 (95% CI: 0.670–0.802) with the sensitivity of 80.6% and the specificity of 59.5%, the cutoff was 0.0175. In summary, the current data supported that exosomal miR-99b-5p and miR-150-5p were the diagnostic biomarkers for early CRC.

### Exosomal miR-99b-5p and miR-150-5p Levels Reflect the Tumor Retention

**Figure 5:**
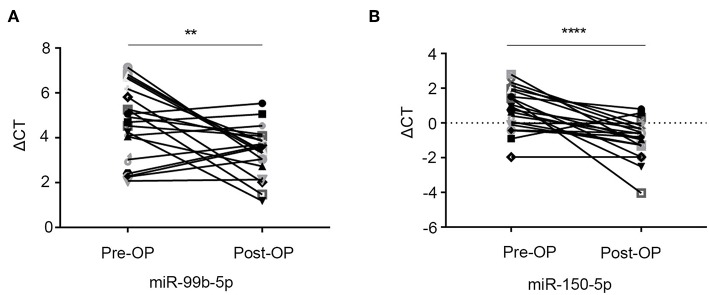
Exosomal miR-99b-5p and miR-150-5p levels reflect tumor retention. **(A,B)** The serum exosomal miR-99b-5p and miR-150-5p levels were significantly higher in the postoperative samples than in the preoperative samples (**p < 0.01, ****p < 0.0001).

To explore the role of exosomal miR-99b-5p and miR-150-5p in CRC, the expression of these molecules was detected in 20 CRC patients who underwent curative surgical resection at pre- and postoperatively. Interestingly, we found that miR-99b-5p and miR-150-5p levels increased markedly after resection of the primary tumors (*p* = 0.0058 and *p* < 0.0001, paired *t*-test, respectively, Figures 5A,B). These results indicated that tumor retention might negatively influence the expression levels of exosomal miR-99b-5p and miR-150-5p, thereby designating it as a novel biomarker to monitor the surgical efficiency.

## Discussion

CRC is one of the most common malignant tumors and the third leading cause of cancer-related deaths worldwide (32). Despite continuous improvements in the treatment, CRC patients are extremely vulnerable to relapse and mortality due to the delayed diagnosis, and thus, sensitive and specific biomarkers are an indispensable requirement for the identification of CRC patients. The current study revealed the critical role of serum exosomal miR-99b-5p and miR-150-5p in CRC, rendering them as promising biomarkers of diagnosis in CRC.

Reportedly, miR-99b-5p and miR-150-5p are involved in several cancers (33, 34). MiR-99b-5p inhibited the proliferation by negatively regulating the insulin-like growth factor 1 receptor (IGF-1R) and activating the AKT signaling pathway in gastric cancer (35). MiR-150-5p suppressed the cell proliferation and invasion in prostate cancer by regulating MAP3K12 (36) and significantly suppressed the aggressiveness of lung squamous cell carcinoma cells. Therefore, miR-99b-5p and miR-150-5p were designated to play a major role in the proliferation and aggressiveness of cancer cells. Moreover, exosomal miR-99b-5p and miR-150-5p might inhibit cancer cell proliferation in CRC serving as putative effective biomarkers for CRC diagnosis.

In this study, the miRNA sequences of two exosomal miRNAs extracted from the serum of CRC patients were analyzed. Several pieces of evidence validated exosomal miR-99b-5p and miR-150-5p as the promising biomarkers for CRC diagnosis. Firstly, exosomal miR-99b-5p and miR-150-5p were remarkably downregulated in CRC patients as compared to healthy donors and benign disease. Secondly, the diagnostic performance of the two miRNAs was assessed. As a result, the AUC of miR-99b-5p was found to be 0.628 with a sensitivity of 32.1% and a specificity of 90.8%, while that of miR-150-5p was 0.707 with a sensitivity of 75.2% and a specificity of 58.8%. Finally, the exosomal miR-99b-5p and miR-150-5p diagnosed the early CRC cancer from healthy donors, and the AUC of miR-99b-5p was 0.652 with the sensitivity of 33.3% and the specificity of 91.5%, while the AUC of miR-150-5p was 0.736 with the sensitivity of 80.6% and the specificity of 59.5%.

Notably, we examined the association between the expression levels of exosomal miR-99b-5p and miR-150-5p and clinicopathological characters and found that the two exosomal miRNAs were associated with gender (*p* = 0.019 and *p* = 0.002, respectively). Previous studies reported that the increased vulnerability of men to developing CRC might be attributed to a number of biological and behavioral factors. Men are likely to have a diet high in red and processed meat, be heavy consumers of alcohol, and be smokers. Also, men have a great propensity to deposit visceral fat, which is associated with an increased risk of CRC (37). Nevertheless, the efficiency of diagnosis did not differ significantly when analyzed in male or female cohorts separately (data not shown). Furthermore, the expression of exosomal miR-99b-5p and miR-150-5p was upregulated after surgery, indicating that tumor retention might negatively influence the expression levels of exosomal miR-99b-5p and miR-150-5p. Taken together, the current data indicated that miR-99b-5p and miR-150-5p were novel biomarkers in CRC diagnostics.

In conclusion, the current data suggested that the levels of exosomal miR-99b-5p and miR-150-5p were significantly downregulated in CRC patients, which was critical for diagnosing CRC patients. Thus, these molecules served as potential clinical diagnostic biomarkers that can accurately and rapidly distinguish the colorectal cancer patients.

## Data Availability Statement

The datasets analyzed in this manuscript are not publicly available. Requests to access the datasets should be directed to l_xie2001@126.com.

## Ethics Statement

The studies involving human participants were reviewed and approved by procedures followed were in accordance with the ethical standards of the responsible committee on human experimentation (Shandong Cancer Hospital Affiliated to Shandong First Medical University and Shandong Academy of Medical Sciences) and with the Helsinki Declaration of 1975. Written informed consent to participate in this study was provided by the participants' legal guardian/next of kin.

## Author Contributions

LX, XinS, and XiaS: guarantor of integrity of the entire study. LX and XinS: study concepts and design. YZ: literature research. YZ and YT: clinical studies. YZ and LN: experimental studies/data analysis and statistical analysis. YZ and LX: manuscript preparation. YZ, LX, and XinS: manuscript editing.

### Conflict of Interest

The authors declare that the research was conducted in the absence of any commercial or financial relationships that could be construed as a potential conflict of interest.
