# Supplementary material for: Circulating Exosomal miR-150-5p and miR-99b-5p as Diagnostic Biomarkers for Colorectal Cancer
Source: Front Oncol. 2019 Oct 23;9:1129. doi: 10.3389/fonc.2019.01129 (PMC6842995; doi:10.3389/fonc.2019.01129)
Supplement: Supplementary file 1 [file Table_1.pdf]

Table S1 ten predicted targets of the selected miR-99b-5p and miR-150-5p

miRNA	Target mRNA	miRNA	Target mRNA
miR-150-5p	MYB	miR-99b-5p	AP1AR
	MDM4		HES7
	ENSA		CTDSPL
	ADIPOR2		NR6A1
	HILPDA		TRIB1
	TADA1		SMARCD1
	MTCH2		KDM6B
	DCAF6		CDYL2
	ELOVL3		IGF1R
	WTAP		SATB1
